# Anti-Vascular Endothelial Growth Factor Therapy as an Alternative or Adjunct to Pan-Retinal Photocoagulation in Treating Proliferative Diabetic Retinopathy: Meta-Analysis of Randomized Trials

**DOI:** 10.3389/fphar.2020.00849

**Published:** 2020-06-05

**Authors:** Shuang Gao, Zhongjing Lin, Xi Shen

**Affiliations:** Department of Ophthalmology, Ruijin Hospital Affiliated Shanghai Jiaotong University School of Medicine, Shanghai, China

**Keywords:** anti-vascular endothelial growth factor therapy, diabetic vitreous hemorrhage, panretinal photocoagulation, proliferative diabetic retinopathy, meta-analysis

## Abstract

**Aim:**

To compare anti-vascular growth factor (anti-VEGF) pharmacotherapy with pan-retinal photocoagulation (PRP) for proliferative diabetic retinopathy (PDR).

**Method:**

PubMed, Embase, Medline, the ClinicalTrials.gov and the Cochrane Central Register of Controlled Trials were reviewed systemically. Randomized controlled trials (RCT) on anti-VEGF therapy versus PRP or anti-VEGF agent combined with PRP versus PRP for PDR are eligible to be included. Outcome measures were regression and recurrence of neovascularization, change in best corrected vision acuity, development of vitreous hemorrhage, and need for vitrectomy. A meta-analysis was conducted using RevMan (Cochrane Collaboration, Oxford, United Kingdom).

**Results:**

Twelve RCTs with a total of 1026 eyes were identified. The meta-analysis results showed that regression of neovascularization did not vary significantly among different treatment regimens (P=0.06), whereas the recurrence of new vessels was significantly lower in PRP monotherapy (P < 0.00001). The best corrected visual acuity was significantly improved with anti-VEGF monotherapy or in the combined group than in the PRP groups (P < 0.00001, P=0.04, respectively). Odds ratio for post-treatment vitreous hemorrhage and vitrectomy rate between anti-VEGF therapy and PRP were 0.65 (95% confidence interval, 0.45–0.95; P = 0.03), and 0.24 (95% confidence interval, 0.12–0.48; P < 0.0001).

**Conclusion:**

Our meta-analysis indicates that anti-VEGF pharmacotherapy is associated with superior visual acuity outcomes and less PDR-related complications. However, there is insufficient evidence to suggest anti-VEGF therapy as an alternative to PRP.

## Introduction

Proliferative diabetic retinopathy (PDR) is the leading cause of blindness, especially among the population of working age. Neovascularization is the key feature of PDR, as vascular endothelial growth factor (VEGF) contributes significantly in the pathologic mechanism of PDR. Effective treatment option can facilitate better visual rehabilitation. Over the past four decades, pan-retinal photocoagulation (PRP) is still the mainstay treatment for PDR (The Diabetic Retinopathy Study Research Group, 1981; Early Treatment Diabetic Retinopathy Study Research Group, 1991) in spite of many unavoidable adverse effects like permanent loss of peripheral vision and aggravation of macular edema. It aims at slowing down the growth of new vessels in the retina extensively (Evans et al., 2014). An over 50% decrease in the probability of severe vision loss was found when PRP was executed on high-risk PDR patients (The Diabetic Retinopathy Study Research Group, 1987). Within 3 months, about 60% of PDR patients respond to PRP with neovascularization (NV) regression (Vander et al., 1991). A survey showed that 98% of retina specialists agreed on choosing PRP for initial PDR management in patients without diabetic macular edema (DME) (American Society of Retina Specialists (ASRS), 2014). Currently, anti-VEGF pharmacotherapy has become a new trend (Simunovic and Maberley, 2015) and already licensed for DME treatment. Whether anti-VEGF drugs could be an adjunctive therapy or even replace PRP for PDR remains an enigma. This is perhaps the first meta-analysis putting emphasis on anti-VEGF as an adjunct or alternative to PRP in PDR.

## Methods

### Search Strategy

We conducted a literature search on PubMed, Embase, Medline, the ClinicalTrials.gov, and the Cochrane Central Register of Controlled Trials, from inception to October 2019. Randomized controlled trials were identified using the following key terms: proliferative diabetic retinopathy, anti-VEGF therapy, panretinal photocoagulation, bevacizumab/avastin, pegaptanib, ranibizumab, aflibercept. Reports of clinical trials were restricted to those making comparison between intravitreal anti-VEGF therapy with or without combination of PRP and PRP in PDR patients. Further, we manually searched the references of these original studies and reviewed potentially relevant articles to supplement the initial search.

### Selection Criteria

Real-world studies of anti-VEGF and PRP therapy for PDR published before search date were all included. The inclusion criteria to screen the captured publications in this meta-analysis were as follows: (1) randomized controlled trials; (2) patients with PDR 18 years and older, who were scheduled for intravitreal anti-VEGF therapy as an alternative or adjuvant treatment to PRP; (3) an observation period of at least 3 months after the treatment. Exclusion criteria were studies with additional interventions, and conditions like previous history of intraocular surgery, anticipated need for vitrectomy within 12 months, or other causes leading to retinal neovascularization. Meeting abstracts, full texts without any raw data for retrieval and review articles were also excluded. If multiple publications were based on the same population, we chose the trial with the largest number of patients.

### Data Extraction

Two reviewers (SG and ZL) independently finished the electronic database search. Data were extracted, and methodological quality of these RCTs was assessed according to the Cochrane Collaboration's “Risk of Bias” tool from the Cochrane Handbook for Systematic Reviews of Intervention (Higgins and Green, 2011). Results were compared and consensus were reached after discussion. We obtained original data from the articles and converted some of the available data into proper form if possible. Considering that the numbers of recruited participants and those actually completed the study are different in some well-designed or large-scale studies, we collected the data according to different follow-up outcomes. Patients with concomitant DME were not taken in to consideration when analyzed the visual outcomes.

### Outcome Measures

Primary outcome measures were anatomical outcomes, including complete regression and recurrence of neovascularization. Secondary outcome measure was the mean change in best corrected vision acuity (BCVA) from baseline to the end of follow-up period. If the mean change in BCVA was available from the original article, we use the data directly, otherwise, we calculated the data according to the following formula (Higgins and Green, 2011): BCVAR = BCVA_baseline_ − BCVA_endpoint_

$${\mathrm{SD}}_{\mathrm{BCVAR}}=\sqrt{{{\mathrm{SD}}_{\mathrm{baseline}}}^{2}+{{\mathrm{SD}}_{\mathrm{endpoint}}}^{2}-{\mathrm{SD}}_{\mathrm{baseline}}\times {\mathrm{SD}}_{\mathrm{endpoint}}}$$

Progression of post-treatment vitreous hemorrhage (PVH) and need for vitrectomy after different therapies were assessed as secondary outcome parameters as well.

### Statistical Analysis

Statistical analyses were done according to Cochrane Collaboration recommendations (PRISMA-P) (Moher et al., 2015). Meta-analysis was performed using RevMan 5.3 software package. We assessed the heterogeneity by using the I^2^ statistic (Higgins and Green, 2011), and regarded I^2^ values of 25%, 50%, and 75% as low, moderate, and high heterogeneity respectively (Higgins and Thompson, 2002). A probability of ≤ 0.05 was considered to be statistically significant for overall effect. Continuous outcomes were calculated using inverse variance analysis with random-effects model and 95% confidence intervals. Proportional outcomes were analyzed using Mantel–Hanszel method and random-effects model with 95% confidence intervals.

## Results

### Included Studies

**Figure 1:**
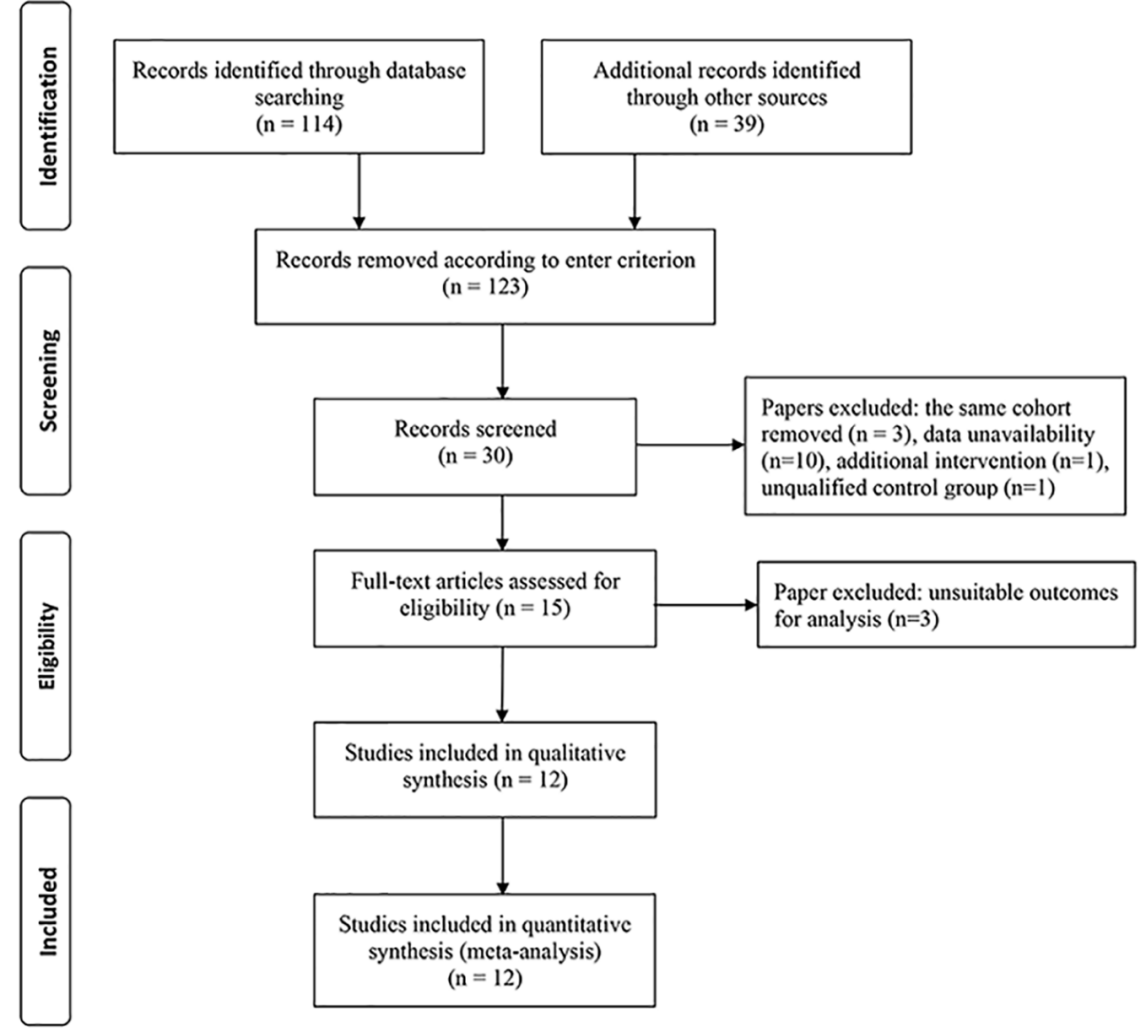
Flowchart of literature search and study selection.

The literature search process is summarized in **Figure 1**. A total of 153 articles were identified, in which 123 articles were excluded based on our selection criteria. However, taking into account the suitable outcomes for performing this meta-analysis, 4 studies (Tonello et al., 2008; Cho et al., 2010; Preti et al., 2013; Diabetic Retinopathy Clinical Research Network, 2013) initially considered relevant were finally excluded. There are a total of 12 RCTs (Mirshahi et al., 2008; González et al., 2009; Cho et al., 2009; Ergur et al., 2009; Filho et al., 2011; Ernst et al., 2012; Messias et al., 2012; Gross et al., 2015; Ferraz et al., 2015; Figueira et al., 2016; Sivaprasad et al., 2017; Figueira et al., 2018), involving 1026 eyes with PDR appraised across all these studies.

### Baseline Characteristics and Risk of Bias

**Figure 2:**
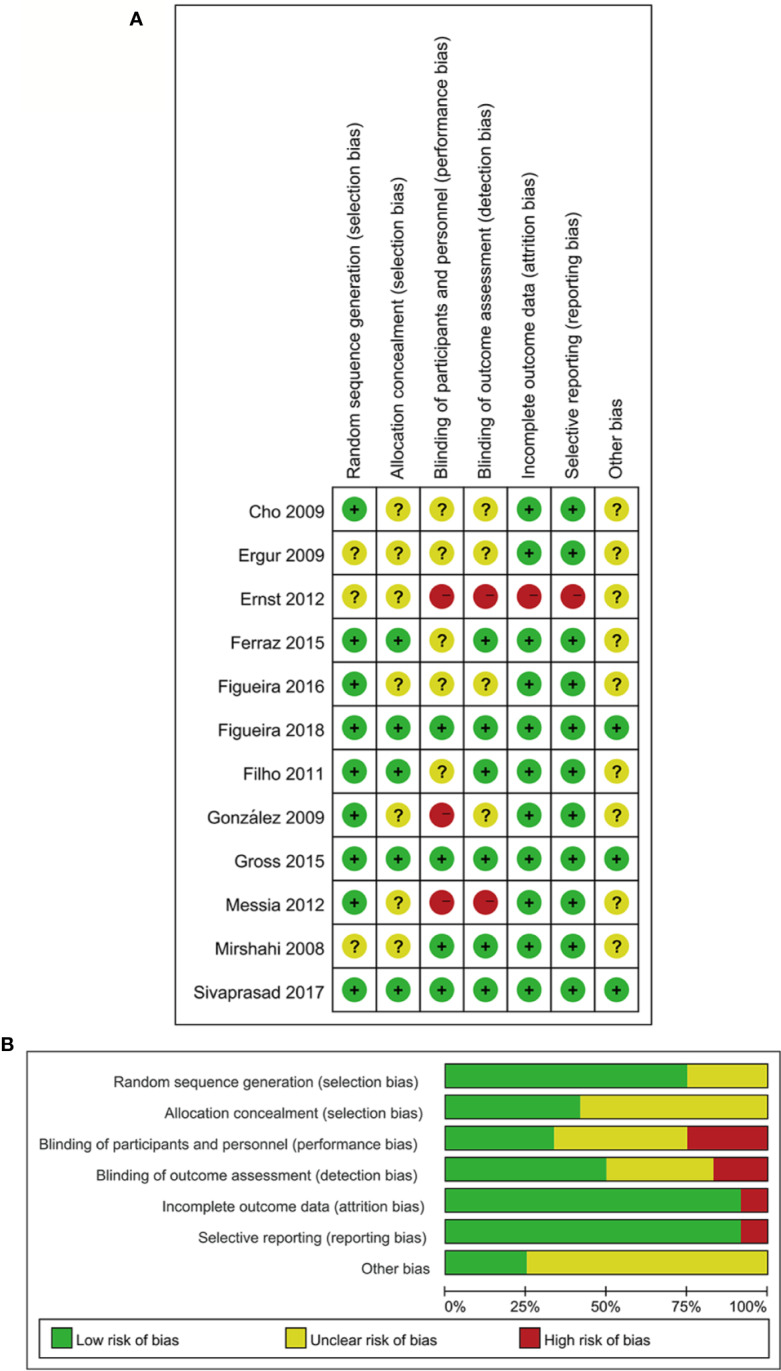
Risk of bias assessment in included studies **(A)**. Risk of bias summary: two review authors' judgments on risk of bias for each included study **(B)** Risk of bias graph: two review authors' judgments presented as percentages on each risk of bias item across all included studies. −, high risk of bias; +, low risk of bias;?, unclear risk of bias.

The characteristics of twelve RCTs are summarized in **Table 1**. With sample sizes varying from 10 to 394 across all the studies, 1026 eyes were pooled from them in order to conduct this meta-analysis. The follow-up duration ranged from 3 months to 2 years. The cumulative sample size of different therapeutic groups comprised 329 with anti-VEGF agent injection, 515 with PRP and 182 with anti-VEGF agent combined with PRP. All these RCTs applied appropriate methods of randomization. Risk of bias for these RCTs is shown in **Figure 2**.

**Table 1:** The characteristics of the included studies in this meta-analysis.

Study	Country	N (eyes)	Intervention	Control	Mean Age (Intervention)	Mean Age (PRP)	Major inclusion criteria	Major exclusion criteria	Primary outcome	Secondary outcome	Number of injections	Length of Follow-up
González et al. (2009)	USA	20	Pegaptanib sodium	PRP	56.2 ± 9.3	59 ± 10.85	High-risk PDR	Hemorrhage/media opacity obscuring fundus visualization	Regression of NVD and/or NVE.	BCVA and CMT	6	36 weeks
Ernst et al. (2012)	USA	10	Ranibizumab	PRP	NA	NA	PDR	DME or prior previous PRP/intraocular surgery	CMT	BCVA	6	12 months
Gross et al. (2015)	USA	394	Ranibizumab	PRP	52 (44, 59)	51 (44, 59)	PDR with VA letter score≥24	Prior PRP	Change in VA letter score	VF total point score, CMT, DME development	10 (6, 13)	2 years
Sivaprasad et al. (2017)	UK	232	Aflibercept	PRP	51.5 ± 14.6	50.8 ± 13.2	Untreated or post-laser treated active PDR	DME or CST ≥ 300 μm	Change in BCVA	Additional visual function and quality-of-life outcomes	4.4 ± 1.7	52 weeks
Figueira et al. (2016)	Portugal	35	Ranibizumab	PRP	61 (52–65)	54 (44–59)	High-risk PDR	Presence of fibrovascular proliferation	New vessel regression	BCVA and CMT	5 (5–7)	12 months
			Ranibizumab+PRP	PRP	57 (49.5–61.5)	54 (44–59)	High-risk PDR	Presence of fibrovascular proliferation	New vessel regression	BCVA and CMT	6 (5–7)	12 months
Mirshahi et al. (2008)	Iran	80	Ranibizumab+PRP	PRP	total 52 (39–68)		High-risk PDR	Prior laser treatment	FFA leakage	NA	NA	16 weeks
Cho et al. (2009)	Korea	41	Ranibizumab+PRP	PRP	61.1 ± 7.8	59.2 ± 8.2	High-risk PDR, and BCVA of 0.3 logMAR	Treatment for DME in previous 3 months; prior PRP	BCVA and CMT	Proportion of visual loss 0.1 logMAR, increase in CMT ≥ 50 μm, VH development	NA	3 months
Ergur et al. (2009)	Turkey	19	Ranibizumab+PRP	PRP	71.4 ± 4.6	68.3 ± 3.4	PDR	Prior laser treatment	BCVA and FLA	NA	NA	6 months
Filho et al. (2011)	Brazil	29	Ranibizumab+PRP	PRP	50.5 ± 3.0	63.3 ± 2.5	High-risk PDR	Prior laser treatment	BCVA	FLA and CMT	1+PRN	48 weeks
Messia et al. (2012)	Brazil	20	Ranibizumab+PRP	PRP	59 ± 12	64 ± 8	High-risk PDR	Prior laser treatment	ERG	BCVA and FLA	1+PRN	48 weeks
Ferraz et al. (2015)	Brazil	60	Ranibizumab+PRP	PRP	53.2 ± 7.7	50.8 ± 7	Non–high-risk PDR with DME	Aphakia, macular ischemia	Change in BCVA and CMT	NA	2	6 months
					53.1 ± 8.7	51.9 ± 8.2	Non–high-risk PDR without DME	Aphakia, macular ischemia	Change in BCVA and CMT	NA	2	6 months
Figueira et al. (2018)	Portugal	87	Ranibizumab+PRP	PRP	58.8 ± 13.3	52.0 ± 11.9	High-risk PDR	DME or CST>300 μm	Regression of NV total	BCVA, CMT, NV recurrence, need for DME treatment need for vitrectomy	Loading phase: 3.0 ± 0.2; follow-up phase: 1.6 ± 1.2	12 months

PDR, proliferative diabetic retinopathy; NVD, neovascularization of the disc; NVE, neovascularization elsewhere; BCVA, best corrected visual acuity; CMT, central macular thickness; NA, not available; DME, diabetic macular edema; VA, visual acuity; VF, visual field; VH, vitreous hemorrhage; ERG, electroretinogram; NV, neovascularization.Number of eyes refers to the number of recruited participate at baseline.

### Efficacy Analysis

#### Regression of Neovascularization

**Figure 3:**
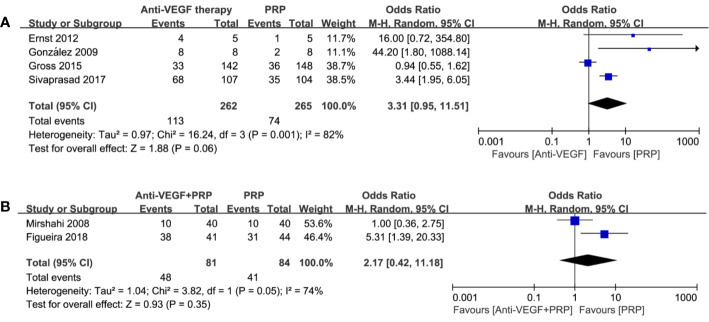
Forest plot comparisons of complete neovascularization regression in patients with PDR after different regimens: **(A)** anti-VEGF monotherapy versus PRP monotherapy, **(B)** combined therapy versus PRP monotherapy. Events: the number of patients with complete neovascular regression.

Complete regression rates of retinal neovascularization at the end of follow-up were obtained, and forest plot comparison is shown in **Figure 3**. The odds ratio for NV regression in 4 studies with relation to anti-VEGF versus PRP was 3.31 (95% CI, 0.95–11.51; P = 0.06; I^2^ = 82%). Analysis of two studies comparing the combined therapy with PRP found that the odds ratio was 2.17 (95% CI, 0.42–11.18; P = 0.35; I^2^ = 74%). There was no significant difference between any of these comparisons, indicating insufficient ability of anti-VEGF drugs to regress NV no matter as an alternative or adjunct to PRP.

#### Recurrence of Neovascularization

**Figure 4:**
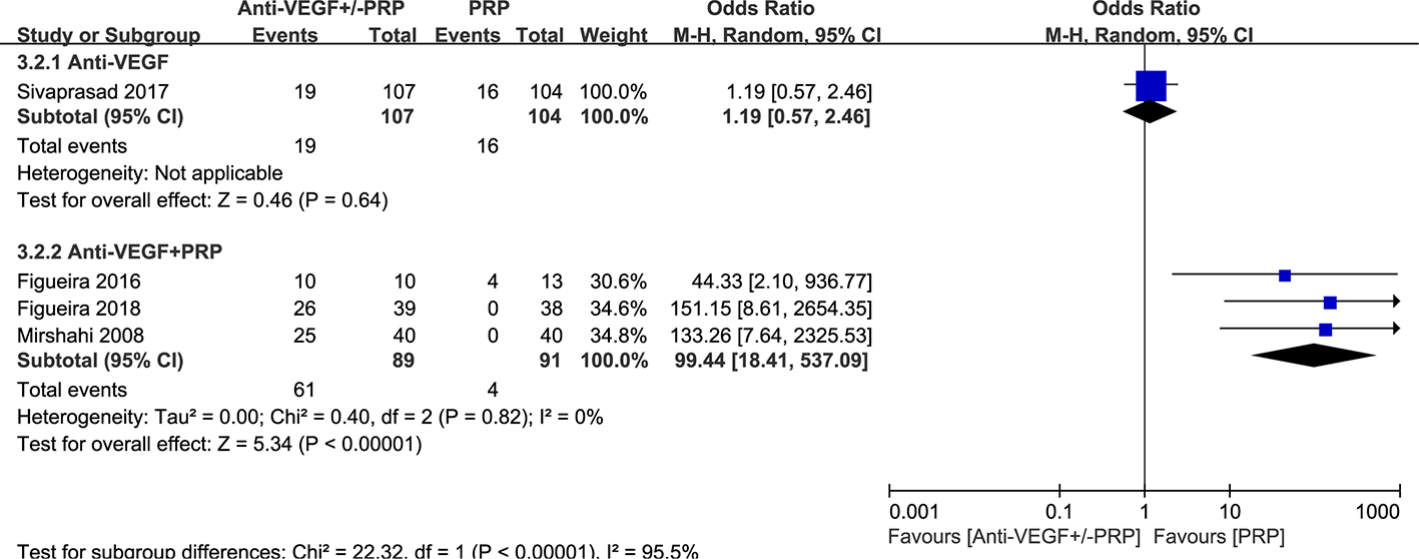
Forest plot comparison between anti-VEGF versus PRP and combined therapy versus PRP monotherapy for the recurrence of neovascularization in patients with PDR. Events: the number of patients with neovascular recurrence.

One study reported the similar recurrence rates between anti-VEGF and PRP groups (P=0.62). Meta-analysis of the incidence of NV recurrence in **Figure 4** suggested that the combined therapy has a much more higher recurrence rate (odds ratio, 99.44; 95% CI, 18.41–537.09; P < 0.00001) in contrast to PRP with no heterogenicity. It is noteworthy that probability of NV reactivation might increase with the application of anti-VEGF therapy, revealing more permanent effect of PRP on NV regression.

#### BCVA

**Figure 5:**
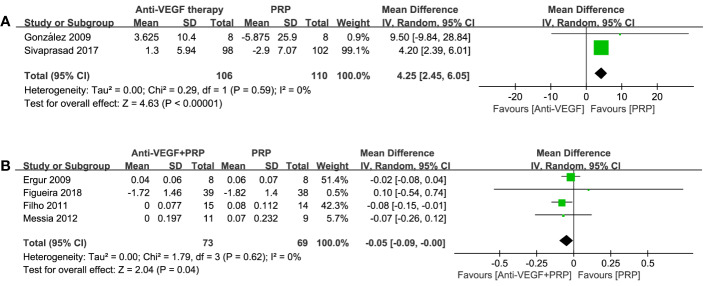
Forest plot comparisons of best corrected visual acuity (BCVA) in patients with PDR after different regimens: **(A)** anti-VEGF monotherapy versus PRP monotherapy (EDTRS), **(B)** combined therapy versus PRP monotherapy (logMAR). Events: change in BCVA from baseline to the last follow-up.

Two studies listed BCVA outcomes using EDTRS at 36 and 52 weeks. Meta-analysis results in **Figure 5** revealed that there was a significant improvement of BCVA in anti-VEGF group compared to PRP (WMD, 4.25; 95% CI, 2.45–6.05; P < 0.00001). When using logMAR to compare the combined group with PRP, a significant advantage of combined therapy was shown (WMD, −0.05; 95% CI, −0.09 to −0.00; P = 0.04), with no heterogenicity identified. The difference between such two comparisons demonstrated that anti-VEGF agents could preferably improve vision rather than PRP monotherapy.

#### Post-Treatment Vitreous Hemorrhage

**Figure 6:**
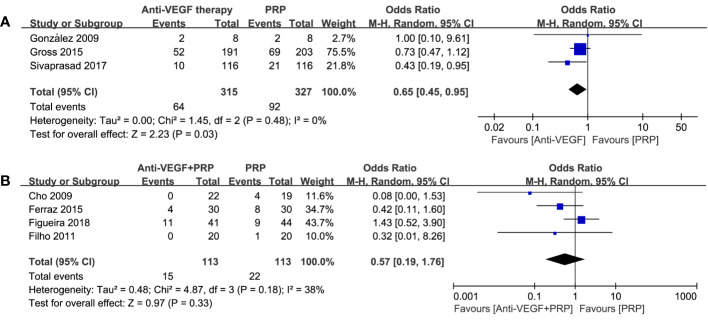
Forest plot comparisons of post-treatment vitreous hemorrhage (PVH) in patients with PDR after different regimens: **(A)** anti-VEGF monotherapy versus PRP monotherapy, **(B)** combined therapy versus PRP monotherapy. Events: the number of patients with PVH.

A total of 7 studies reported the incidence of PVH. The pooling results manifested that there was a significant difference between anti-VEGF therapy and PRP (odds ratio, 0.65; 95% CI, 0.45–0.95; P=0.03) with no heterogenicity, while combined therapy was not significantly different compared to PRP (odds ratio, 0.57; 95% CI, 0.19–1.76; P = 0.33; I^2^ = 38%). Detailed results could be seen in **Figure 6**. Thus, we considered anti-VEGF monotherapy to be an effective approach to reduce the incidence of PVH. Combined treatment may be rarely worth trying under certain circumstance.

#### Post-Treatment Vitrectomy Rate

**Figure 7:**
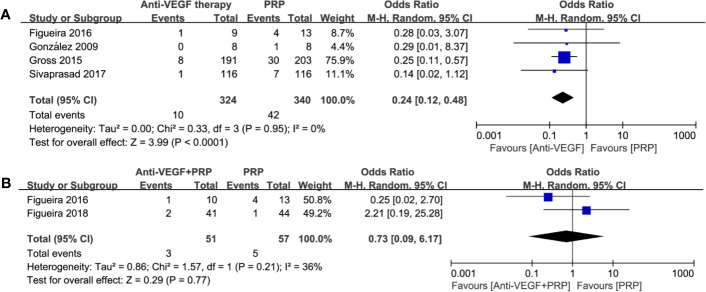
Forest plot comparisons concerning the incidence of post-treatment vitrectomy in patients with PDR. **(A)** anti-VEGF monotherapy versus PRP monotherapy, **(B)** combined therapy versus PRP monotherapy. Events: the number of patients requiring vitrectomy.

4 studies comparing anti-VEGF therapy with PRP were extractable. Forest plot comparison in **Figure 7** indicated obvious benefit of anti-VEGF therapy with no heterogenicity (odds ratio, 0.24; 95% CI, 0.12–0.48; P < 0.0001) on lowering post*-*treatment vitrectomy rate during the follow-up period. However, combined therapy did not show an advantage on reducing the need for vitrectomy (odds ratio, 0.73; 95% CI, 0.09–6.17; P = 0.77; I^2^ = 36%). Such findings were similar to PVH, which remind us of the clinical potential of anti-VEGF monotherapy for preventing post-treatment vitreous hemorrhage and vitrectomy.

## Discussion

Quality of Evidence from RCTs considering the safety and efficacy of anti-VEGF agents for PDR used to be low (Martinez-Zapata et al., 2014). Currently, several RCTs somewhat covers this shortage (Gross et al., 2015; Ferraz et al., 2015; Sivaprasad et al., 2017; Figueira et al., 2018). In this meta-analysis, we failed to find a better response in terms of total NV reduction in anti-VEGF pharmacotherapy combined with or without PRP. Meanwhile, PRP showed its great advantage in prevent NV recurrence. Occurrence of PVH or any PDR-related complications requiring vitrectomy was rarer in eyes assigned to anti-VEGF monotherapy. Anti-VEGF therapy appears to play a role as primary treatment.

Anti-VEGF therapy is believed to have promising neovascular regression outcomes in active PDR patients (Avery, 2006) though its duration is limited. The CLARITY non-inferiority RCT (Sivaprasad et al., 2017) considered evaluation of anatomical effect to be an important standard when choosing the first-line option for PDR. It reported superior NV regression capacity of aflibercept, but a 2-year study (Gross et al., 2015) found no statistically significant difference in this outcome between ranibizumab and PRP. Many studies demonstrated superior short-term anatomical effects of anti-VEGF agents in combination with PRP for high-risk PDR patients (Mirshahi et al., 2008; Yang et al., 2013; Simunovic and Maberley, 2015; Zhou et al., 2016). Typically, Mirshahi et al. (2008) reported dynamic neovascular changes of combined and PRP group in week 6 and week 16. Complete regression could be induced in 87.5% of Avastin-injected eyes, over 3 times that of PRP group at week 6. However, new vessels recurred in Avastin-treated eyes at week 16 of follow-up, and the regression rate in the two groups became equal. Meanwhile, several studies (Jorge et al., 2006; Tonello et al., 2008; Filho et al., 2011; Arevalo et al., 2017) demonstrated that PRP combined with or without anti-VEGF therapy could not reach full regression of NV, either in treatment-naïve patients or patients with persistent NV after PRP. The latest PROTEUS study (Figueira et al., 2018) also found that complete NV regression was induced more rapidly in combined treatment group than PRP group at 12 months follow-up (3.6 and 7.0 months, respectively). Nevertheless, recurrence rate reached 67% in the combined group approximately, while no reactivation of new vessel was found in patients treated with PRP. Recurrence of NVs could begin as early as 2 weeks after anti-VEGF agent injection (Avery, 2006). Research on pharmacokinetics of ranibizumab revealed an aqueous half-life of 7.19 days (Krohne et al., 2012). Half-life of anti-VEGF agents might be its major shortcoming when compared with the durability of PRP. Interestingly, it is reported that approximately 30% of patients with PDR were unable to respond to initial anti-VEGF treatment (Bromberg-White et al., 2013). One possible explanation is that increased VEGF levels might be sufficient to overcome the initial anti-VEGF therapy. In other words, VEGF expression is alternatively regulated in PDR. Repeated intravitreal anti-VEGF injection may play the key role in sustaining a steady anatomical outcome (Schmidinger et al., 2011). In summary, anti-VEGF therapy is still in its infancy from the perspective of anatomical changes. The combined approach has shown its great potential in revolutionizing the management of high-risk PDR in spite of the permanent effect of a complete PRP treatment. For patients with high-risk PDR, complete PRP within the effective period of anti-VEGF agents might be recommended.

It cannot be neglected that weighing the relative benefits of PRP and anti-VEGF agents in PDR treatment is influenced by the presence of DME. Probability of vison-impairing DME development is lower in anti-VEGF group (Gross et al., 2018). Bressler et al. (2017) found that cumulative probability of worsening PDR was 34% (ranibizumab) versus 42% (PRP; P = 0.063) through 2 years. For those eyes accompanied with center-involved DME, this rate was higher with PRP than ranibizumab (45% vs 31%; P=0.008). If managing PDR with PRP, risk of DME development and poor vision improvement increased with higher hemoglobin A1c level and worse level of baseline PDR severity (Bressler et al., 2018). However, Gross et al., (2018) proceeded to do a 5-year visit and regarded ranibizumab or PRP as two viable treatments for PDR, indicating PRP did no harm to DME treatment with the presence of ranibizumab.

Several RCTs showed no significant improvement in visual acuity with the application of anti-VEGF agents (Tonello et al., 2008; Ergur et al., 2009; Messias et al., 2012; Figueira et al., 2018). However, according to a Cochrane Review (Martinez-Zapata et al., 2014), PDR patients who received anti-VEGF therapy had better visual acuity than those who received PRP alone at follow-up. In this meta-analysis, anti-VEGF therapy combined with or without PRP was found to provide better visual acuity than PRP monotherapy, indicating that anti-VEGF agent is a feasible new approach for BCVA improvement. Nevertheless, the follow-up durations of these studies were no more than 48 weeks, reminding us that researches on long-term outcomes are necessary. No difference was identified between the two treatment regimens for PDR in most of the patient-centered outcomes (Beaulieu et al., 2016). The unique 5-year study reported the similar outcome of visual acuity in most eyes of PRP and anti-VEGF monotherapy (Gross et al., 2018).

Secondary to PDR, vitreous hemorrhage (VH) can cause severe vision loss. A Cochrane Review (Smith and Steel, 2015) revealed that incidence of early postoperative vitreous cavity hemorrhage was lowered after pre- or intraoperative bevacizumab. Our meta-analysis also demonstrated an obvious benefit of anti-VEGF monotherapy for lowering VH rate. Nevertheless, once the combined therapy was used, the benefit is no longer clear. Need for vitrectomy due to the occurrence of PDR-related complications was investigated as well. The cumulative incidence of vitrectomy at the end of follow-up period was rather higher in PRP group than anti-VEGF monotherapy (Ernst et al., 2012; Gross et al., 2015; Figueira et al., 2018). Approximately half of the eyes in ranibizumab and PRP groups developed VH over 5 years (P = 0.47). Vitrectomy was performed in 41% and 22% of the eyes in PRP and ranibizumab group, respectively (P = 0.008) (Gross et al., 2018). It follows that combined therapy might not work better than anti-VEGF monotherapy on reduction of VH and vitrectomy rate.

Patients with multiple diabetes comorbidity, low compliance, and treatment fatigue are also the obstacles to overcome (Ting and Wong, 2017). Lin et al. (2018) conducted a decision analysis in order to assess cost and cost-utility of PRP and intravitreal ranibizumab (IVR) for PDR without DME. For 2 years of utility in facility setting, modeled cost per quality-adjusted life years of treatment was $163 988 in PRP group and $436 992 in IVR group. Another retrospective cohort study (Obeid et al., 2018), with 2303 patients enrolled, reported a higher rate of loss to follow-up (LTFU) in PRP group, which may be explained by its more durable effect, higher pain level (Lucena CRF de et al., 2013) and some complications. Obeid et al., 2019 (Obeid et al., 2019) further investigated 59 PDR patients who were LTFU for over 6 months, in which anti-VEGF monotherapy manifested worse anatomic and functional outcomes. Wei et al. (2017) also reported that fibronectin and fibrinogen concentrations in vitreous humor of intravitreal anti-VEGF injection group, which might promote fibrosis in eyes with PDR. Thus, many problems of anti-VEGF therapy remain to be solved. These may limit the enthusiasm for intensive treatment regimen of anti-VEGF drugs.

Several limitations are unavoidable. First, the limited number of RCTs, especially high-quality large-scale RCTs, made our results less convincing. Second, the time points of these extracted data were inconsistent, varied from 3 months to 2 years. Many studies found that a large amount of PDR patients showed a recurrence of neovascularization beginning at 3 months (Arevalo et al., 2009; Schmidinger et al., 2011), so we chose an observation period of at least 3 months after the initial treatment. Third, different treatment protocols were used in these included RCTs. Fourth, patients with coexisting DME may be separately analyzed if more large-scale clinical trials are available. Fifth, the degree of PDR severity was not accurately quantified. Sixth, comparison could not be made among different anti-VEGF agents. Actually, several questions are still unknown as well, like the average frequency of intravitreal injections, proper timing of injection and right choice among various branded anti-VEGF agents (Gross and Glassman, 2016). Although two RCTs (Gross et al., 2015; Sivaprasad et al., 2017) have established intravitreal ranibizumab and aflibercept as noninferior, or even superior treatment options for PDR, different branded anti-VEGF agents vary considerably in their affinity and half-life, which correlate directly with clinical effect. Innovation of anti-VEGF agent delivery assumes a vital role in the future.

## Conclusions

In conclusion, it is necessary to further assess the long-term visual, anatomical, and safety outcomes of anti-VEGF therapy. PRP is still irreplaceable in the field of PDR. Innovation of anti-VEGF agent delivery assume a vital role in the future. Our results may provide some insights into weighing the pros and cons among anti-VEGF agents, PRP and combined therapy in PDR treatment. Individualized treatment relying on patient's baseline condition, compliance level, and economic status is the most important part in promoting therapeutic effect and optimizing vision care. It remains to be elucidated whether anti-VEGF agent is a time-tested beneficial therapy like PRP.

## Data Availability Statement

The raw data supporting the conclusions of this article will be made available by the authors, without undue reservation, to any qualified researcher.

## Author Contributions

XS: study design, reviewed the manuscript. SG and ZL: database management and search strategies, meta-analysis, wrote, and reviewed the manuscript.

## Funding

Science and Technology Commission of Shanghai Municipality (No.18411964900), Shanghai Municipal Commission of Health and Family Planning (No.201740004) supported this work.

## Conflict of Interest

The authors declare that the research was conducted in the absence of any commercial or financial relationships that could be construed as a potential conflict of interest.
